# Postglacial genetic legacies and climate-driven demography inform conservation of silver fir

**DOI:** 10.1093/aob/mcag029

**Published:** 2026-02-09

**Authors:** Francisco Balao, Marc Ríos-Cadenas, José Manuel Sánchez-Robles, María Teresa Lorenzo, Juan C Linares, Anass Terrab

**Affiliations:** Departamento de Biología Vegetal y Ecología, Facultad de Biología, Universidad de Sevilla, Sevilla 41012, Spain; Departamento de Biología Vegetal y Ecología, Facultad de Biología, Universidad de Sevilla, Sevilla 41012, Spain; Departamento de Biología Vegetal y Ecología, Facultad de Biología, Universidad de Sevilla, Sevilla 41012, Spain; Departamento de Didáctica de las Ciencias Experimentales, Facultad de Ciencias de la Educación, Universidad de Granada, Granada 18071, Spain; Departamento de Biología Vegetal y Ecología, Facultad de Biología, Universidad de Sevilla, Sevilla 41012, Spain; Departamento de Sistemas Físicos, Químicos y Naturales, Universidad Pablo de Olavide, Sevilla 41013, Spain; Departamento de Biología Vegetal y Ecología, Facultad de Biología, Universidad de Sevilla, Sevilla 41012, Spain

**Keywords:** *Abies alba*, margin, phylogenomics, range, recolonization routes, RAD-seq

## Abstract

**Background and Aims:**

This study explores the genetic diversity and past range dynamics of silver fir (*Abies alba*) with the objective of delineating the past demography of the species, the main postglacial colonization pathways and the current vulnerability of past glacial refuges.

**Methods:**

We applied restriction site-associated DNA sequencing (RAD-seq) to 26 *A. alba* populations to explore their phylogeographic structure and historical demographic changes

**Key Results:**

Three lineages were identified. One comprised populations from the southern range, whereas northern populations formed two lineages with subtle genetic differentiation between western and eastern populations. Northern Balkan and north-eastern Apennine samples clustered within introgression zones at the intersection of postglacial recolonization routes, consistent with their intermediate geographic positions. Demographic analyses indicated that southern populations remained relatively stable as glacial refugia, whereas northern populations experienced population declines during the middle Pleistocene associated with glaciations. Although the demographic events that shaped the current spatial structure of genetic diversity of silver fir go back to the Pleistocene, human pressure during the Holocene likely led to an abrupt range decline.

**Conclusions:**

We present a timely and broadly applicable framework for delineating the phylogeographic lineages of silver fir, leveraging the high resolution provided by SNP markers to identify major genetic discontinuities, contact zones and refugial areas. Inferences of past demographic dynamics indicate that certain rear-edge populations warrant priority in conservation strategies.

## INTRODUCTION

While climate change constrains the persistence of numerous tree species (Allen *et al.*, 2015; McDowell *et al.*, 2020), the biogeography and genetic diversity of populations could be key to achieving local adaptations (Aitken *et al.*, 2008). From a biogeographical perspective, taxon responses to environmental change are likely shaped by population dynamics at range margins, where rear-edge populations may act as long-term reservoirs of genetic diversity (Davis and Shaw, 2001; Hampe and Petit, 2005). Further, the relationship between s**t**anding genetic diversity and adaptive phenotypic characteristics may be essential to cope with climate change (Barrett and Schluter, 2008; Alberto *et al.*, 2013).

Range-wide patterns of population genetic diversity are related to past climate-driven range dynamics. In the Northern Hemisphere, this standing genetic diversity was strongly influenced by post-glacial gene flow (Hewitt, 2000, 2004; Davis and Shaw, 2001). Hence, tree populations have been subject to cyclical patterns of extinction and dispersal, driven by the dynamics of glacial periods. During glaciations, forest species contracted their ranges, taking refuge in southern disjunct refugia, and subsequently extended their range northwards in response to climatic amelioration during interglacial periods of the Pleistocene (Hewitt, 1999). The major Mediterranean peninsulas are considered to have served as glacial refugia for temperate tree populations, whereas more northern areas have also been proposed as refugia for certain boreal and temperate European forest taxa (Tzedakis *et al.*, 2002; Svenning *et al.*, 2008; Médail and Diadema, 2009). These refugia have significantly influenced the genetic structure and diversity of areas recolonized after the glaciations (Hewitt, 2000). Northern refugia of temperate species were identified by the fossil record (e.g. fir in Pannonia or Moravia,Willis and van Andel, 2004), but often there is no evidence of their trace in the current gene pools. Precise identification of glacial refugia is essential for conservation strategies, as these locations might retain the core of species’ genetic diversity (Hewitt, 2004; Keppel *et al.*, 2012; Fady *et al.*, 2022). Furthermore, these areas are crucial for the long-term preservation of biodiversity and face threats from rapid environmental changes in the Mediterranean area (Sánchez-Salguero *et al.*, 2017). However, the precise identification of these refugia, and consequently the delineation of postglacial migration routes, remains elusive for certain species due to inherent methodological challenges (Willis and Van Andel, 2004).

Firs (*Abies*, Pinaceae) are among the dominant trees of the temperate-cool forests of the Northern Hemisphere (Farjon and Rushforth, 1989). A recent study focusing on the evolutionary history of the Mediterranean firs supports the idea that all Mediterranean species form a single, monophyletic group (Balao *et al.*, 2020). Furthermore, this research provides evidence of two lineages within the Mediterranean firs, which align with the sections previously identified as *Abies* and *Piceaster* (Farjon and Rushforth, 1989). Additionally, this study suggests an early diversification of the Mediterranean *Abies* species (late Oligocene to Early Miocene) coupled with both ancient and recent admixture resulting from secondary contacts. This underscores the crucial role of historical climatic events in the speciation process and genomic architecture of the species in this fir lineage. Among the Mediterranean firs, silver fir (*Abies alba*) is the sole species with a widespread range, whereas other species (e.g. *A. pinsapo*, *A. numidica*, *A. nebrodensis*) are generally confined to narrow or highly restricted ranges (Linares, 2011). *Abies alba* currently inhabits Central Europe and there are patent populations in the Pyrenees, southern and eastern Alps, the Carpathians and the Dinaric Alps, which are connected to the Rhodope Mountains. It is also present in the Massif Central and in the Apennines (EUFORGEN, 2011).

**Fig. 1.**
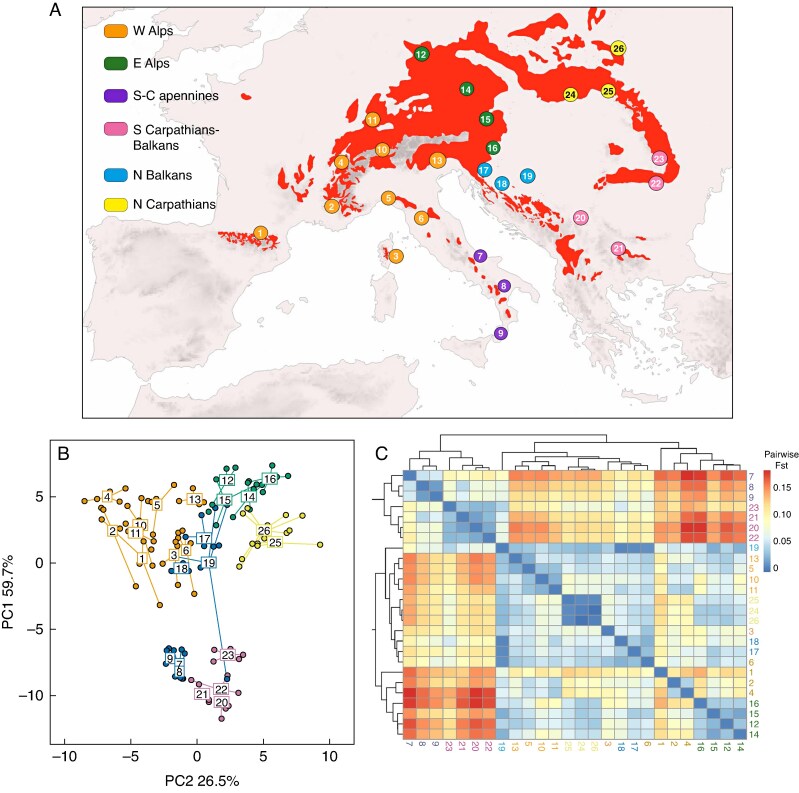
(A) Map showing sampled populations within the distribution range of *Abies alba*, marked in red (Source: EUFORGEN). Numbers indicate the investigated population locations (detailed in Table 1), with the main geographic regions differentiated by colour. (B) PCA revealing regional segregation across 26 populations along north–south and west–east axes. (C) Heat map displaying *F*_ST_ genetic distances; warmer colours signal greater distances, cooler colours lesser distances.

The glacial and postglacial history of silver firs is based on palaeoecological data and extensive molecular research conducted only at the regional level or using a limited number of genetic markers (e.g. Fady *et al.*, 1999; Hussendörfer, 1999; Vendramin *et al.*, 1999; Longauer, 2001; Gömöry *et al.*, 2012; Piotti *et al.*, 2017; Teodosiu *et al.*, 2019; Kempf *et al.*, 2020; Scotti-Saintagne *et al.*, 2021; among others), with the exception of Liepelt *et al.* (2009), which sampled along the distribution range and used two sets of markers. During the Last Glacial Maximum, *A. alba* is hypothesized to have found refuge primarily in two principal locations, the northern Apennines and the southern Balkan Peninsula (Fig. 1). Further, refugia in the southern Apennines and the Pyrenees have been proposed (Liepelt *et al.*, 2002, 2009; Terhürne-Berson *et al.*, 2004; Litkowiec *et al.*, 2016), although these populations might have remained largely isolated, resulting in only limited expansions and consequently making them genetically distinct (Di Pasquale *et al.*, 2014; Piotti *et al.*, 2017; Scotti-Saintagne *et al.*, 2021). Nevertheless, the Pyrenees refugium has been considered uncertain by other authors (Reille and Lowe, 1993). Additionally, palaeogeological evidence also suggests different ice-age refugia of silver fir in two major glacial refugia located in the northern Apennines and north-western Greece (Cheddadi *et al.*, 2014).

The potential pathways for postglacial recolonization and zones of introgression for *A. alba* have also been under debate (Konnert and Bergmann, 1995; Liepelt *et al.*, 2002). It is proposed that the recolonization of north-western, central and north-eastern European regions occurred via two primary postglacial routes: (1) originating from the central Apennines, and (2) emanating from the southern Balkan Peninsula (Fig. 1). The northward colonization from the northern Apennines would occur via two subroutes, as suggested by Kral (1980). In the ‘western Alpine route’, silver fir moves along the Ligurian mountains into the south-eastern French Alps and then colonizes the Jura, Vosges mountains and Black Forest. The ‘eastern Alpine route’ outflanks the Alps and leads to the Thuringian Forest. The second major colonization route linked the southern Balkan Peninsula silver fir populations to northern areas via two additional subroutes. A ‘western Balkan route’ would move along the Dinaric Alps to the eastern Alps. The ‘eastern Balkan route’ would colonize the Carpathians via southern Bulgaria. Although most studies have outlined the importance of range shifts (Williams *et al.*, 2004), the potential for genetic responses has often been neglected, and so very few studies (Martínez-Sancho *et al.*, 2021) have thoroughly investigated the genomic and demographic consequences of glacial cycles throughout the full distribution range of silver fir, to validate these proposed refugia and postglacial migration routes.

Compared with previous studies on this taxon, which were based on a more limited set of markers and/or partial geographic coverage, this study uses a robust high-throughput sequencing approach (RAD-seq) to genotype thousands of loci, providing precise estimates of genetic divergence, diversity patterns and spatial population structure. This is combined with a range-wide sampling scheme (26 populations) encompassing the entire distribution of the species, allowing us to characterize broad-scale spatial genetic patterns that could not be resolved previously. Our approach is fundamentally aimed at testing key hypotheses concerning the principal postglacial colonization pathways and the past demography of the species that have shaped the present-day distribution of silver fir. We specifically aimed to (1) delineate the phylogenomic relationships among populations of silver fir, (2) identify and characterize genetic discontinuities and contact zones between distinct genetic lineages, thereby elucidating the landscape genetic structure, (3) investigate the historical population size changes and how range dynamics have shaped current patterns of genetic variation, and (4) discuss conservation priorities from the inference of past demography and current vulnerability to climate change.

## MATERIALS AND METHODS

### Sample collection and DNA extraction

Due to the wide distribution of silver fir in western and central Europe and its history of cultivation on the continent, the populations included in this study were carefully selected. Samples were collected *in situ* from presumably natural stands during several field trips in 2014–16, using bibliographic resources and geographic information from the EUFORGEN project. We prioritized populations located within national parks or other protected areas. In total, we sampled 26 populations, aiming to cover the entire distribution range of the species across 14 countries (six in Italy; three in Croatia and France; two in Germany, Poland and Romania; and one in Austria, Bulgaria, Czech Republic, Serbia, Slovak Republic, Slovenia, Spain and Switzerland; Fig. 1A and Table 1). In each population, leaves offive (four) adult treeswere collected and preserved in silica gel (126 individuals in total). Sample sizes within this range have been shown to yield reliable estimates of population divergence and spatial genetic structure, provided that a sufficient number of SNPs (>1000) is analysed (Nazareno *et al,* 2017; Cao *et al,* 2020; among others). In all cases, we sampled the oldest available trees, ensuring they were spaced sufficiently far apart (20–30 m) to minimize the likelihood of collecting closely related individuals. Genomic DNA was extracted from leaf material stored in silica gel using a DNA extraction kit (Qiagen DNeasy Plant Mini Kit) following the manufacturer’s instructions. The RAD library was prepared as described in Balao *et al.* (2020). Genomic DNA was digested by using high-fidelity SbfI (New England Biolabs) and the resulting fragments were double-barcoded. The library was sequenced in a separate lane of an Illumina flow cell HiSeq 2500 at the VBCF NGS Unit (www.vbcf.ac.at/ngs) as 100-bp single-end reads.

**Table 1.** The 26 *Abies alba* populations sampled. *N* represents the number of individuals analysed.

Population	Location	Region	Country	Altitude (m)	Coordinates	*N*
1	Valencia de Aneu	Pyrenees	Spain	1500	42°38′N, 01°04′E	5
2	Station de Lure, Provence Alps and Prealps	South-western Alps	France	1600	44°06′N, 05°49′E	5
3	Col de Verde	Central Corsica	France	966	42°02′N, 09°11′E	5
4	Saint Laurent, Savoy Prealps	North-western Alps	France	1044	46°01′N, 06°20′E	5
5	Favale di Malvaro, western Emilian Apennines	Northern Apennines	Italy	950	44°28′N, 09°14′E	5
6	Badia Prataglia, eastern Tuscan Apennines	Northern Apennines	Italy	980	43°47′N, 11°52′E	5
7	Castiglione, southern Abruzzi Apennines	Central Apennines	Italy	1000	41°44′N, 14°18′E	4
8	Laurenzana, Lucan Apennines	Southern Apennines	Italy	1200	40°25′N, 15°58′E	4
9	National Park Aspremonte, Calabrian Apennines	Southern Apennines	Italy	993	38°15′N, 16°01′E	5
10	Prato, Lepontine Alps, Swiss Prealps	North-western Alps	Switzerland	1358	46°28′N, 08°45′E	5
11	Häusern	Southern Black Forest	Germany	630	47°43′N, 08°12′E	5
12	Rauensteim	Thuringian Forest	Germany	500	50°24′N, 11°02′E	5
13	La Valle Agordina, Dolomites	Southern limestone Alps	Italy	900	46°16′N, 12°04′E	5
14	Boubin virgin forest, Gratzen Mountains	Southern Bohemia	Czech Republic	990	48°57′N, 13°48′E	5
15	Rothwald, Ybbstal Alps, Northern Lower Austria Alps	North-eastern Alps	Austria	750	47°44′N, 15°03′E	5
16	Puščava, Northeastern Slovene Prealps	South-eastern Alps	Slovenia	430	46°32′N, 15°25′E	5
17	National Park Risnjak	Northern Dinaric Alps	Croatia	700	45°25′N, 14°39′E	5
18	National Park Plitvice Lakes	Northern Dinaric Alps	Croatia	576	44°52′N, 15°36′E	4
19	Park Prirode Papuk, Slavonia	Papuk Mountains	Croatia	480	45°31′N, 17°31′E	5
20	Mt Goč	Kopaonik Mountains	Serbia	1060	43°33′N, 20°49′E	5
21	Rila Monastery Nature Park	Rila-Rhodope Massif	Bulgaria	1260	42°08′N, 23°22′E	5
22	Sinaia, Bucegi Mountains	Southern Carpathians	Romania	770	45°18′N, 25°33′E	4
23	Bǎile Tusnad, Harghita Mountains	South-eastern Carpathians	Romania	669	46°08′N, 25°51′E	5
24	Poprad, Kozie Chrbty Mountains	North-western Carpathians	Slovak Republic	770	49°00′N, 20°16′E	5
25	Bieszczadzki Park Narodowy, Eastern Beskids	North-eastern Carpathians	Poland	820	49°06′N, 22°47′E	5
26	Obrocz, Roztocze National Park	North-eastern Carpathians	Poland	272	50°35′N, 23°03′E	5

### RAD-seq catalogue building and SNP calling

Data processing was performed using the following workflow. (1) Quality filtering and demultiplexing of the library were performed with deML (Renaud *et al.*, 2015) and STACKS v2.55 (Rochette *et al.*, 2019). Low-quality reads (Q < 30) and reads without the restriction cut site were discarded. (2) For SNP calling and genotyping, reads were mapped using Bowtie 2 (Langmead and Salzberg, 2012) to the published *A. alba* draft genome (Mosca *et al.*, 2019) using the –very-sensitive mode. Reads with mapping quality <30 and coverage >200 were discarded, and PCR duplicates were marked using SAMtools 1.16.1 (Danecek *et al.*, 2021). The genome-aligned reads were assembled into RAD loci using ref_map.pl code implemented through STACKS and the populations STACKS pipeline was used to export read data into various formats for subsequent analyses. We retained biallelic loci that exhibited a presence in a minimum of 70 % of individuals and with a minor allele frequency (MAF) >0.05 across the entire dataset. In the subsequent analyses we used a 22 019-SNP dataset.

### Population genetic diversity

Basic population genetic statistics were assessed and described using standard population genetic parameters. Using STACKS v2.55, and for each population, we estimated the number of private alleles, number of polymorphic sites, expected heterozygosity (*H*_e_), observed heterozygosity (*H*_o_), inbreeding coefficients (*F*_is_) and nucleotide diversity (*π*) with their standard errors. Based on phylogeographic criteria commonly applied to European tree distributions and on previous work in silver fir (Konnert and Bergmann, 1995; Piotti *et al.*, 2017; Teodosiu *et al.*, 2019), we defined regional groupings to provide a geographic framework for interpretation. Populations from the south-western (SW) region were assigned to the southern–central (S–C) Apennines, whereas those from the south-eastern (SE) region were grouped as southern (S) Carpathians–Balkans. Within the northern range, the north-eastern (NE) region was subdivided into northern (N) Balkans, N Carpathians and the eastern (E) Alps, while north-western (NW) populations were assigned to the western (W) Alps (Fig. 1A).

### Phylogenomic analyses and genetic structure

A maximum likelihood phylogeny was inferred using RAxML-ng v. 1.10 (Stamatakis, 2014). The analysis was conducted under the GTR + GAMMA model and Felsenstein’s ascertainment-bias correction (conditioning the likelihood on variable sites only) was applied to account for SNP ascertainment. To mitigate this bias, the + ASC_FELS parameter was employed, with a specific value (802 204) denoting the invariable sites that were incorporated into the analysis. Branch support values were computed from 1000 bootstrap replicates. To situate the evolutionary position and as a point of reference for understanding the evolutionary relationships, we included five individuals of *Abies cephalonica* from three Greek populations (Oros Ainos, Oros Pateras and Kalavryta; for details see Balao *et al.*, 2020) as outgroups in the phylogenetic tree.

A principal component analysis (PCA) was estimated using the R package adegenet (Jombart and Ahmed, 2011) in order to determine the genetic structure of populations. Additionally, we performed a Bayesian assignment analysis with ADMIXTURE version 1.3.0 (Alexander *et al.*, 2009) using the 22 019-SNP dataset. We conducted ADMIXTURE analysis by running the software with different levels of grouping (*K*), ranging from 1 to 26. For each level of grouping, we performed 100 replicates. The best-fitting *K* was chosen based on the estimated cross-validation (CV). Additionally, alternative *K* values were assessed to explore potential refinements in structural resolution.

### Isolation by distance and geographic patterns of genetic diversity

The extent of genetic differentiation among various locations was quantified using the fixation index (*F*_ST_). To calculate pairwise *F*_ST_ values, we employed the R package StAMPP (Pembleton *et al.*, 2013). A matrix of *F*_ST_ scores was generated for each pair of populations under investigation. To visualize genetic structure, a heat map was constructed, accompanied by a Euclidean distance dendrogram. To examine the relationship between genetic differentiations (represented by *F*_ST_) and geographic separation, we conducted an isolation by distance (IBD) analysis to explore the impact of geographic distance on the genetic differentiation across all 26 populations. This was accomplished by gauging the correlation between genetic differentiation (*F*_ST_/(1 − *F*_ST_)) and geographic distance (log-transformed) through the utilization of the R package dart (Gruber *et al.*, 2018). To investigate the potential relationship between genetic diversity estimators and geographic variables, we also formulated various geographically explicit generalized linear models (GLMs) using both genetic and geographic data. We considered the nucleotide diversity (*π*) and the number of private alleles as the dependent variables, with latitude and longitude as independent variables. The relationship between private alleles and geographic coordinates was analysed using a GLM with a Poisson distribution as the link function. In contrast, to assess the correlation between *π* and geographic coordinates, we applied a GLM with a Gaussian distribution.

### Population splits and migration topology

TreeMix version 1.13 (Pickrell and Pritchard, 2012) was employed to analyse linkage disiquilibrium-pruned data, with the aim of inferring gene flow events among the 26 populations. For these analyses, we also removed missing data from the dataset, resulting in a 4821-SNP dataset. In these analyses, we compared the likelihood from one to five migration events (*m*). Each analysis included 500 bootstrap replicates, with population 20 used as the outgroup. This outgroup was selected from the four S Carpathians populations that were closest to *A. cephalonica* in the phylogenetic reconstruction (Supplementary Data Fig. S1).

### Demographic reconstructions

We studied the *A. alba* past demography, for the three lineages (S, NW and NE) obtained in the ADMIXTURE analysis, by inferring the effective population size (*N*_e_) over time using the composite likelihood approach with a multi-epoch model implemented in the Stairway Plot v2.1.1 software. We identified loci out of Hardy–Weinberg equilibrium using VCFtools and discarded sites with evidence of heterozygote excess. We polarized the SNPs using one *A. cephalonica* individual sampled from the Oros Pateras population (for details see Balao *et al.*, 2020) as an outgroup. Then, we estimated the site frequency spectrum (SFS) from 18 512 SNPs by employing easySFS (accessible at https://github.com/isaacovercast/easySFS) to mitigate the impact of incomplete data and optimize the overall count of SNPs using a hypergeometric projection of the dataset. Stairway Plot was run considering a generation time of 40 years and assuming a mutation rate of 1.23 × 10^−8^ per site per generation per locus (Mauri *et al.*, 2016; De La Torre *et al.*, 2017). We used the recommended (nseq − 2) ÷ 4 break points (where nseq is the number of haplotypes sampled) and 200 replicate runs for each population.

## RESULTS

The 100 bp Illumina RAD-seq for the 126 samples (1.31 million reads on average per sample) representing all the 26 silver fir populations produced in the final STACKS catalogue a total of 779 282 RAD loci with an average coverage per sample of 34.3 ± 14.2 (s.d.) reads per locus. We found 6.62 % of missing RAD loci among samples, with a mean of 1458.19 ± 63.81. After retaining only polymorphic RAD loci that were present in at least 88 individuals and had a maximum coverage of 100 reads per locus, we obtained a final data set with 8711 RAD loci and a total of 22 019 SNPs.

### Gene diversity

The analysis of genetic diversity (Table 2) showed that the S populations exhibited the highest number of private alleles (518.57 ± 73.17). In these populations, the highest average values of private alleles were found in the S–C Apennines (661.33 ± 87.82) and the S Carpathians–Balkans (411.5 ± 77.24). The S–C Apennine and S Carpathian–Balkan populations showed the highest values of nucleotide diversity, which ranged from 0.095 to 0.102. In general, the N populations showed a lower number of private alleles (189.16 ± 22.5). Furthermore, the N populations displayed a lower average genetic diversity (*π* = 0.087) in contrast to the S populations (*π* = 0.098). The lower nucleotide diversity values (≤ 0.081) were found in the W and E Alps (Table 2). The *F*_is_ values were homogeneous (from −0.007 to 0.045) and did not exhibit statistically significant differences when examined across population groups and geographic locations (Table 2).

**Table 2.** Genetic diversity of the 26 *Abies alba* populations and regional averages.

Region	Subregion	Population	Polymorphic alleles	Private alleles	*H*_o_	*H*_e_	*π*	*F*_IS_
North-western								
	Western Alps	1	62.9	332	0.087 ± 0.001	0.074 ± 0.001	0.085 ± 0.001	−0.002 ± 0.005
	Western Alps	2	60.7	130	0.084 ± 0.001	0.073 ± 0.001	0.083 ± 0.001	−0.001 ± 0.005
	Western Alps	3	68.2	223	0.097 ± 0.001	0.082 ± 0.001	0.094 ± 0.001	−0.007 ± 0.005
	Western Alps	4	57.1	117	0.077 ± 0.001	0.070 ± 0.001	0.079 ± 0.001	0.004 ± 0.004
	Western Alps	5	63.9	102	0.085 ± 0.001	0.076 ± 0.001	0.085 ± 0.001	0.002 ± 0.003
	Western Alps	6	70.7	275	0.094 ± 0.001	0.082 ± 0.001	0.093 ± 0.001	−0.001 ± 0.005
	Western Alps	10	61.8	115	0.087 ± 0.001	0.074 ± 0.001	0.084 ± 0.001	−0.006 ± 0.004
	Western Alps	11	63.4	168	0.089 ± 0.001	0.076 ± 0.001	0.087 ± 0.001	−0.005 ± 0.005
	Western Alps	13	62.6	147	0.084 ± 0.001	0.075 ± 0.001	0.085 ± 0.001	0.002 ± 0.003
**Mean ± standard error**			**63.5 ± 1.3**	**178.8 ± 26.9**	**0.087** ± **0.002**	**0.076** ± **0.001**	**0.086** ± **0.002**	**−0.002 ± 0.001**
North-eastern								
	Eastern Alps	12	59.5	89	0.077 ± 0.001	0.072 ± 0.001	0.081 ± 0.001	0.009 ± 0.003
	Eastern Alps	14	61.0	167	0.077 ± 0.001	0.074 ± 0.001	0.083 ± 0.001	0.013 ± 0.004
	Eastern Alps	15	67.1	418	0.091 ± 0.001	0.079 ± 0.001	0.089 ± 0.001	−0.001 ± 0.004
	Eastern Alps	16	57.4	62	0.077 ± 0.001	0.071 ± 0.001	0.080 ± 0.001	0.008 ± 0.003
	Northern Balkans	17	69.8	324	0.081 ± 0.001	0.081 ± 0.001	0.091 ± 0.001	0.023 ± 0.004
	Northern Balkans	18	63.1	223	0.093 ± 0.001	0.078 ± 0.001	0.093 ± 0.001	0.000 ± 0.004
	Northern Balkans	19	74.4	301	0.074 ± 0.001	0.084 ± 0.001	0.094 ± 0.001	0.045 ± 0.004
	Northern Carpathians	24	65.8	128	0.088 ± 0.001	0.076 ± 0.001	0.086 ± 0.001	−0.002 ± 0.004
	Northern Carpathians	25	66.0	138	0.089 ± 0.001	0.078 ± 0.001	0.088 ± 0.001	−0.001 ± 0.004
	Northern Carpathians	26	65.6	135	0.088 ± 0.001	0.077 ± 0.001	0.087 ± 0.001	−0.002 ± 0.004
**Mean ± standard error**			**65.0 ± 1.6**	**198.5 ± 36.4**	**0.083** ± **0.002**	**0.077** ± **0.001**	**0.087** ± **0.002**	**0.009** ± **0.005**
South-western								
	Southern–Central Apennines	7	63.0	530	0.095 ± 0.001	0.08 ± 0.001	0.095 ± 0.001	0.001 ± 0.005
	Southern–Central Apeninnes	8	68.8	626	0.099 ± 0.001	0.085 ± 0.001	0.101 ± 0.001	0.003 ± 0.004
	Southern–Central Apennines	9	77.2	828	0.094 ± 0.001	0.087 ± 0.001	0.100 ± 0.001	0.012 ± 0.005
**Mean ± standard error**			**69.7 ± 4.1**	**661.3 ± 87.8**	**0.096** ± **0.002**	**0.084** ± **0.002**	**0.099** ± **0.002**	**0.005** ± **0.003**
South-eastern								
	Southern Carpathians–Balkans	20	70.8	370	0.091 ± 0.001	0.083 ± 0.001	0.095 ± 0.001	0.008 ± 0.006
	Southern Carpathians–Balkans	21	78.3	638	0.104 ± 0.001	0.089 ± 0.001	0.102 ± 0.001	−0.004 ± 0.005
	Southern Carpathians–Balkans	22	65.8	346	0.099 ± 0.001	0.083 ± 0.001	0.098 ± 0.001	−0.001 ± 0.004
	Southern Carpathians–Balkans	23	72.9	292	0.096 ± 0.001	0.084 ± 0.001	0.096 ± 0.001	0.000 ± 0.005
**Mean ± standard error**			**71.9 ± 2.6**	**411.5 ± 77.242**	**0.098** ± **0.003**	**0.085** ± **0.001**	**0.098** ± **0.002**	**0.001** ± **0.003**

### Phylogenomic analyses and genetic structure

The PCA (Fig. 1B) revealed a pronounced N–S geographic structure and, to a lesser extent, differentiation between NE and NW populations. The first principal component (PC1) explained 59.7 % of the total variance and separated populations into two primary groups corresponding to the N and S regions. The second principal component (PCA2), which accounted for the 26.5 % of the variance, delineates the separation between W and E populations, both in the N and S regions. The phylogenetic tree (Supplementary Data Fig. S1) was partially congruent with this phylogeographic structure, a basal clade represented populations from the SE range of the species and includes populations from the S Balkans and the populations from the S and SE Carpathians. The second grouping, which was greatly differentiated from the other groups and resulted in the most related to the first one, represented the SW populations and includes the populations from the S and C Apennines. The third major group clustered all the N populations.

### Geographical patterns of diversity and IBD

**Fig. 2.**
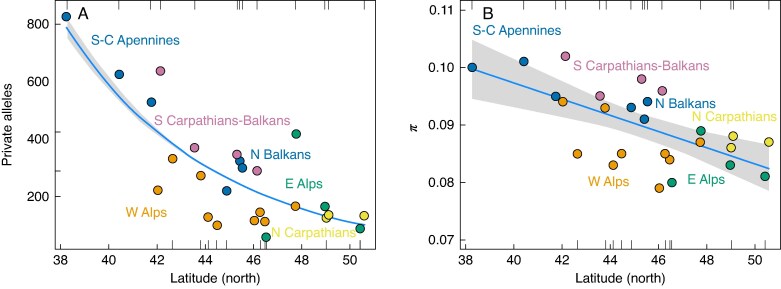
Latitudinal variation in genetic diversity of the *Abies alba* populations. (A) Private alleles. (B) Nucleotide diversity (*π*). Colour denotes the main geographic areas.

Notably, the highest population pairwise *F*_ST_ values were observed between the S populations (from the S–C edna Apennines and S Carpathians–Balkans) with the NW (from W Alps) and NE (from the E Alps and N Carpathians) populations (Fig. 1C). In contrast, genetic differentiation among the S populations appeared to be minimal. Interestingly, the three populations in the N Carpathians region exhibited nearly identical genetic profiles, suggesting a possible recent divergence event. Furthermore, we found a significant relationship between the genetic diversity estimators (*π* and private alleles) and latitude. The analysis clearly indicated that the S region, particularly the S–C Apennines and the S Carpathians–Balkans, demonstrated a significantly higher presence of private alleles (with *P*-values for private alleles and *π* both being <0.001; Fig. 2A). Conversely, regions such as the N Carpathians and E Alps displayed a comparatively diminished presence of private alleles. Additionally, an inverse relationship was observed between latitude and the prevalence of private alleles; their frequency decreased northwards across the geographical region. This trend was consistently mirrored in the genetic diversity values (Fig. 2B), showing a decreasing gradient of genetic diversity with increasing latitude. In contrast, the S region consistently exhibited elevated genetic diversity values. Furthermore, GLM analyses also showed a significant relationship between genetic diversity estimators (*π* and private alleles) with longitude (for private alleles, *χ*^2^ = 1866.1, *P* < 0.001 and for *π*, *χ*^2^ = 16.662, *P* < 0.001; Supplementary Data Fig. S2), with a clear upward trend in both private alleles and genetic diversity (*π*) as populations move further eastward. Among these populations, those belonging to the S Carpathians–Balkans region exhibit the highest levels of genetic diversity and display a significant number of private alleles. Conversely, populations located farther west, such as those in the W Alps region, showed comparatively lower values. An exception to this trend was observed in the N Carpathians region, where, despite its eastern location, lower values were recorded.

### Population coancestry and migration

**Fig. 3.**
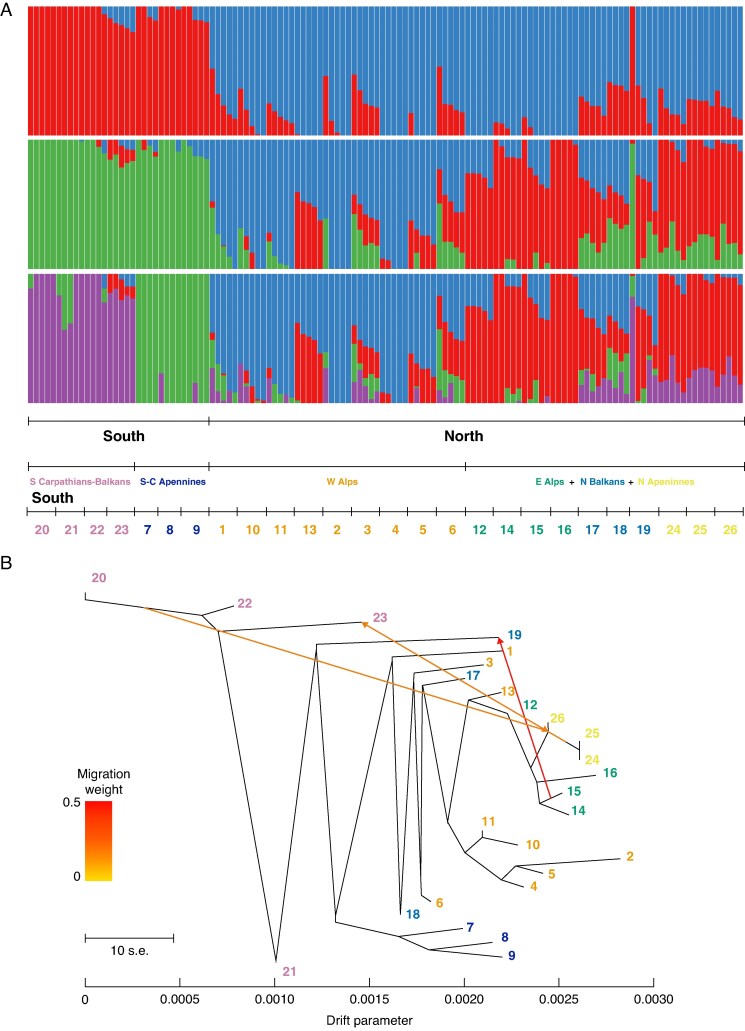
ADMIXTURE analysis revealing genetic clusters among 26 silver fir populations: *K* = 2 separates them into northern and southern groups; *K* = 3 splits the northern group into NE and NW; *K* = 4 resolves four clusters: NE, NW, SE and SW. (B) Maximum likelihood population graph inferred by TreeMix representing the principal migrations between the 26 populations. Results for *m* = 3 showed the three principal migrations. Arrows indicate inferred gene flow events, arrow direction follows the fitted migration edge and line colour/width is proportional to the estimated migration weight.

Admixture results (Fig. 3A) revealed that *K* = 3 showed the best fit with the lowest CV error (Supplementary Data Fig. S3) which discerned the S, NW and NE populations. The green cluster corresponded to all S populations originating from the S–C Apennines and the S Carpathians–Balkans region. The blue cluster represents populations located in the NW. Lastly, the red cluster encompassed populations from E Alps and N Balkans (NE). A clear admixture signal of the three clusters was found in the SW Alps and N Balkans populations (Supplementary Data Fig. S4). In addition, we explored *K* = 2 and *K* = 4 (which showed comparable CV error; Fig. 3A), which exhibited clear differentiation into two main clusters (N–S) and four subclusters, corresponding to both N–S and E–W divisions.

The TreeMix results (Fig. 3B) illustrate migration patterns between populations from different regions. Based on the log-likelihood, the best-supported TreeMix model inferred a single migration edge (*m* = 3; log-likelihood = 813.7) from Romanian populations in S Carpathians to the N Carpathians populations. This introgression was also supported for *m* = 2–5. In the model incorporating two migration events, it was additionally observed that the ancestral populations from Switzerland and Germany in the W Alps had interactions with other W Alps populations. Furthermore, *m* = 3–5 analyses suggested introgression between Croatian populations in the N Balkans and Serbian populations in the S Carpathians–Balkans. A fourth putative migration (*m* = 4–5) would have occurred from Italian populations in the S–C Apennine to Bulgarian populations in the S Carpathians–Balkans. Lastly, we observed an additional introgression (*m* = 5) between W Alps populations, specifically Italian and Pyrenean populations.

### Demographic reconstructions

**Fig. 4.**
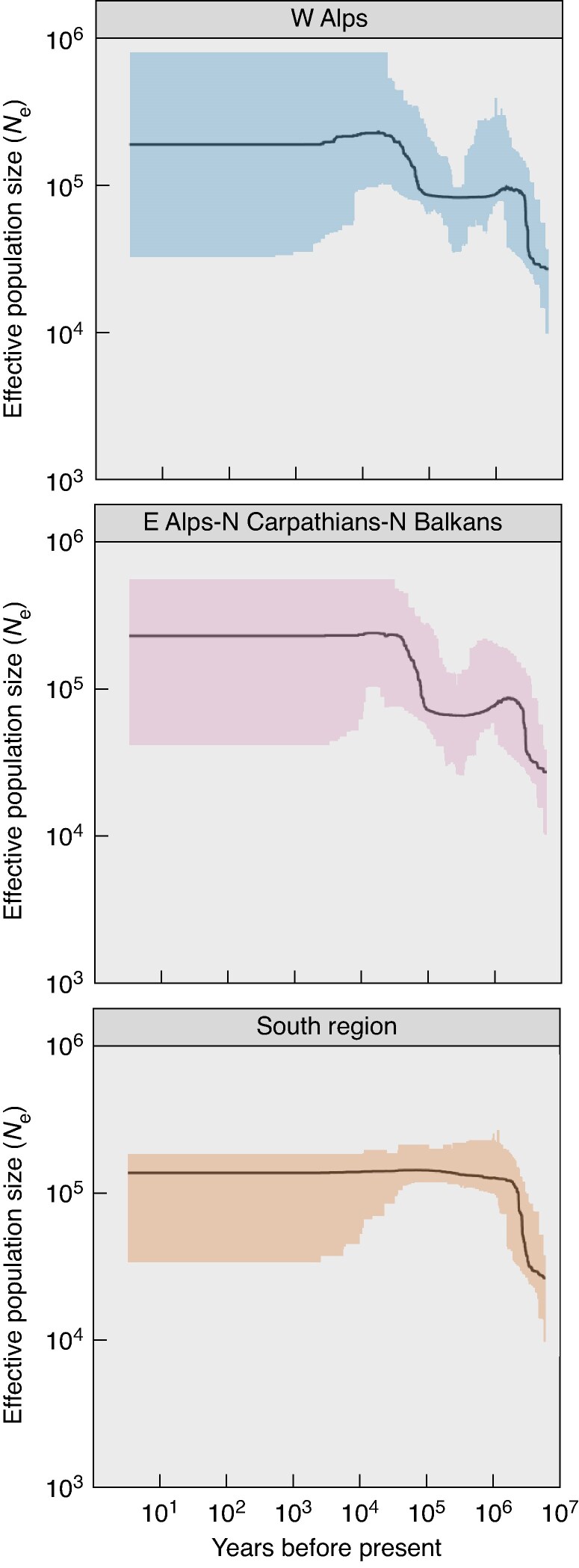
Demographic history of *Abies alba* populations via stairway plot 2 illustrating changes in effective population size (*N*_e_) over the past 5 million years up to the present. Time is scaled by generation time (*g* = 40 years) and mutation rate (*μ* = 1.23 × 10^−8^ substitutions per site per generation). The solid line represents the estimated effective population size; shaded area denotes the 95 % confidence interval.

Demographic analyses of the three genetic lineages (S, NW and NE), identified through the ADMIXTURE analysis, revealed differences in the dynamics of effective population size (*N*_e_) over time between the northern (NW and NE) and southern (S) genetic lineages (Fig. 4). The NW and NE genetic lineages exhibited similar demographic trajectories, both marked by a pronounced decline in *N*_e_, beginning around 1 million years ago and continuing until ∼100 000 years ago. Around 80 000 years ago, these regions experienced a rapid exponential growth in *N*_e_, followed by a stable phase that persisted until roughly 20 000 years ago. Conversely, the S populations (southern genetic lineage) did not undergo such a pronounced decline during the Middle Pleistocene (Fig. 4). They exhibited a gradual increase in population size over time, followed by a gradual decline during the Late Pleistocene that has continued to the present. Specifically, during the last 50 000 years, the S populations have shown a continuous decline (Supplementary Data Fig. S5), with the rate of decrease notably intensifying ∼5000 years ago. In contrast, the demographic trajectories of the N populations have been markedly different. These populations showed growth during the last glaciations, persisting until ∼20 000 years ago, at which juncture a gradual decline began. Notably, this decline appears also to have accelerated from 5000 years ago onwards.

## DISCUSSION

### Landscape genetic structure

Our genome-wide analysis revealed a clear N–S differentiation and a moderate E–W geographic gradient. These findings are not only supported by multiple regional studies across the distribution of *A. alba* but also synthesize and unify the disparate pieces of evidence that have been emerging, providing a comprehensive biogeographic and genetic overview of the species. Regarding the Apennine populations, significant genetic divergence between the C and S populations (SW populations) and those from the NW populations was observed, as demonstrated by allozyme and microsatellite markers (Parducci *et al.*, 1996; Piotti *et al.*, 2017). Interestingly, our results highlight a historical trans-Adriatic connection between silver fir populations in the central and southern Apennines (SW populations) and those in the Balkans and S Carpathians (SE populations). This connection is supported by genetic similarities across several molecular markers, as evidenced in previous studies (Liepelt *et al.*, 2002; Longauer *et al.*, 2003; Piotti *et al.*, 2017). Notably, the SE lineage emerges as the most ancestral group in the phylogram, positioned closer to *Abies cilicica* samples, which supports the hypothesis of an eastern origin for the species (Linares, 2011; Balao *et al.*, 2020). Moreover, within the Carpathians a distinct genetic differentiation is evident, segregating the SE populations from those in the NE and W Carpathians, a pattern also noted by Gömöry *et al.* (2012) using mitochondrial markers.

For the two genetic lineages identified in the northern range (NE and NW), the ADMIXTURE analysis supported the differentiation of the westernmost populations from the Pyrenees, NW and SW Alps (continental French and Swiss populations), north-westernmost Apennines and that from the S Black Forest, from those of the eastern part, clustering populations from the NE and NW Carpathians, and also populations from the SE and NE Alps, S Bohemia and Thuringian Forest. A parallel W–E geographical pattern was observed using mtDNA markers, pinpointing a contact zone between these two phylogeographical lineages in Croatia, Slovenia and NE Italy, a finding that was similarly identified by Breitenbach-Dorfer *et al.* (1997). Regarding the origin of populations in the SW Alps, our findings align with those presented by Sagnard *et al.* (2002), which observed no clear geographical structure among these populations, nor did they detect IBD. Together, these patterns point to substantial historical gene flow among populations and support the hypothesis that SW Alpine populations likely derive from a shared glacial refugium, probably located in the Ligurian Apennines.

The placement of the Corsican population in our analysis revealed ambiguity. Phylogenetic analysis positioned it between the N Balkans and Apennines, whereas PCA suggested closer affinity to the populations from the SW French Alps, S Italian Alps and the Pyrenees. Palynological evidence from Reille *et al.* (1999) indicated that the migration of the species to the island occurred only during the late Holocene, adding another layer of complexity to its historical biogeography.

Finally, although certain studies had previously considered the Pyrenees population to have been isolated since the last glacial period (Konnert and Bergmann, 1995), or even earlier (Scotti-Saintagne *et al.*, 2021), most of our results included the Pyrenees population within the NW populations (PCA, ADMIXTURE; Figs 1B and 3A and Supplementary Data Fig. S4). Although a cultivated origin for this population cannot be excluded, a genetic relationship between the specific Pyrenean population sampled here and a NW French Alps population has been documented by Fady *et al.* (1999). Nonetheless, expanding the sampling in the Pyrenees would be valuable for gaining a deeper understanding of the connections between Pyrenean populations and those in the NW part of the species’ range.

### Legacies of the Holocene colonization

Our results revealed a pronounced eastward increase in nucleotide diversity and private alleles, with the S Carpathians–Balkans populations exhibiting the highest diversity, while W populations, such as those in the Western Alps, showed lower values. These patterns align with previous studies highlighting E refugial persistence during Pleistocene glacial cycles (e.g. Belletti *et al.*, 2017; Teodosiu *et al.*, 2019) and mirror trends reported in other European conifers (*Pinus* spp.; Vendramin *et al.*, 2008). The enrichment of private alleles in E populations likely reflects long-term demographic stability and limited gene flow, in contrast to W populations, which appear to have experienced stronger bottlenecks and founder effects. Comparative analyses with other southern European tree species, including *Fagus sylvatica* and *Quercus* spp., further indicate that species-specific life-history traits and dispersal capacities modulate genetic responses to historical climate fluctuations. Collectively, these findings support a model in which E glacial refugia have disproportionately contributed to postglacial recolonization, thereby shaping the contemporary genetic landscape of *A. alba* across Europe.

Correspondingly, observed patterns of genetic diversity together with phylogenomic and population structure analyses indicate that silver fir survived in different S refugia throughout the last glaciations. Our data strongly confirm the existence of a glacial refugium in the S Apennines (most probably in Aspromonte, S Calabrian Apennines), which appears to have remained isolated from the N Apennines refugia. Consequently, these populations experienced limited expansions and have remained genetically distinct from those in the N Apennine chain (see also the following section, Conservation setting from demography and vulnerability to ongoing climate change on demography). Although the existence of an effective glacial refugium of silver fir in the S or SE Balkans is undoubtedly given by our results (populations with high gene diversity and private alleles), its precise location has remained unresolved due to the scarcity of fossil records (Liepelt *et al.*, 2009), In contrast, our genetic results clearly point to the Rila–Rhodope Massif, or a nearby region, as the most likely refugial area. However, due to the genetic relationship of the W and NE Carpathians populations, another refugium from the N Balkan Peninsula (most likely in N Croatia) cannot be discarded. Lastly, based on the intermediate position of the N Balkans populations, we propose that silver fir from Apennine and Balkan refugia had met and developed in two introgression zones, one in NW and NE Croatia and probably in the foothills of the Slovenian Alps, and a second in High Tatra, Slovak Carpathians, where silver fir from the S Balkan area had met silver fir from N Apennine during its migration.

This intricate biogeographic history contrasts markedly with that of certain comparable tree species, such as Norway spruce (*Picea abies*), which underwent extensive northward and eastward range expansions across Europe following the Last Glacial Maximum, recolonizing substantial portions of Scandinavia and Central Europe from S refugial populations (Tollefsrud *et al.*, 2008). Here, the presence of relatively stable refugia indicates that certain silver fir populations have persisted in suitable habitats throughout Quaternary climatic oscillations to the present, conferring exceptional biogeographic significance and high conservation value (Tzedakis *et al.*, 2002; Hampe and Petit, 2005). Furthermore, some of these relict silver fir populations, such as those from the S Apennines, might have not been the source of major postglacial recolonizations, thereby preserving high genetic distinctiveness.

### Conservation setting from demography and vulnerability to ongoing climate change

Although the main demographic events that shaped the current spatial structure of genetic diversity of silver fir go back to the Pleistocene, human pressure during the Holocene likely led to further population decline (Büntgen *et al.*, 2014). Besides, recent drought events have caused widespread decreasing productivity and dieback of silver fir forest, mainly over its southernmost range limits (Cailleret *et al.*, 2014; Gazol *et al.*, 2015; García-García *et al.*, 2025). In our study, S populations showed a comparatively stable trajectory until the late Pleistocene (∼100 000 years ago), acting as glacial refuges (Linares, 2011). Subsequently, our analyses indicate a continuous population decline over the past 50 000 years, likely driven by factors such as rising minimum temperatures, increased drought stress or competition with more cold-tolerant species (Di Pasquale *et al.*, 2014; Gazol *et al.*, 2015). Anthropogenic pressures appear to have exacerbated this trend, contributing to a rapid demographic decline over the last 5000 years (Linares *et al.*, 2011), a pattern similar to that observed in *Abies pinsapo* in the southern Iberian Peninsula (Leuschner and Ellenberg, 2017; Sánchez-Robles *et al.*, 2022). Notably, a distinct pattern is evident in N regions, where populations that had been expanding began experiencing a reduction in population size during the middle Pleistocene (from ∼1 000 000 years ago to around 200 000 years ago) due to glaciations in this era (Liepelt *et al.*, 2009; Linares *et al.*, 2011). Northern populations likely experienced exponential growth after the last glacial period, aligning with the colonization and expansion from glacial refugia of S populations migrating northwards in the late Pleistocene and the onset of the Holocene (Liepelt *et al.*, 2002, 2009; Terhürne-Berson *et al.*, 2004; Litkowiec *et al.*, 2016). As the S regions, the N populations also suffered an abrupt decline ∼5000 years ago, which would correspond with the impact of human pressures (Leuschner and Ellenberg, 2017). Dendroecological studies confirm contrasting growth responses across the species range, with S Apennine populations exhibiting higher resilience and recovery than W Alpine populations during drought events (Carrer *et al.*, 2010; Oggioni *et al.*, 2024). Similar patterns of decline and mortality are reported in SE European mountains and Mediterranean ecotones, reflecting the combined effects of climate and anthropogenic pressures (Macias *et al.*, 2006; Diaci *et al.*, 2011; Linares and Camarero, 2012; Cailleret *et al.*, 2014).

In this regard, international conservation efforts coordinated through the EUFORGEN programme have proposed up to 298 conservation units across the species distribution range (https://www.euforgen.org/species/abies-alba; de Vries *et al.*, 2015). Our analyses of genetic structure and diversity show that, in most cases, the proposed units match the main genetic patterns identified. However, an important gap remains in the conservation of *A. alba*: the S Carpathians populations, which harbour high diversity and private alleles, are not covered by the current proposal. Although countries in the S distribution of *A. alba* (Romania, Italy and Serbia) have made notable efforts, we recommend the inclusion of new genetic conservation units in three key areas: the Rila–Rhodope Massif (Bulgaria), the Laurenzana region in the Lucanian Apennines (Italy) and the Kopaonik Mountains in southern Serbia.

Our results underscore the importance of incorporating phylogeographic and demographic insights into the conservation planning of silver fir. Populations located in long-term glacial refugia have acted as reservoirs of ancestral diversity and have contributed disproportionately to postglacial recolonization dynamics. Conserving these populations is therefore essential to maintaining the evoluti nary legacy, genetic distinctiveness and adaptive potential of the species throughout its range.

## Supplementary Material

mcag029_Supplementary_Data

## Data Availability

The scripts and data used in this project are publicly available in a Zenodo repository (DOI:10.5281/zenodo.18587991). All demultiplexed RAD-seq raw reads have been deposited in the NCBI Sequence Read Archive under BioProject PRJNA1242184. Sampling was conducted in compliance with Nagoya Protocol regulations in each respective country.

## SUPPLEMENTARY DATA

Supplementary data are available at *Annals of Botany* (https://academic.oup.com/aob) and consist of the following. Figure S1: phylogenetic tree of 126 tree samples from 26 *Abies alba* populations, constructed via maximum likelihood using RAxML-ng. Colours denote main geographical regions. Figure S2: GLM analysis of genetic diversity vs longitude in *Abies alba* populations: (A) private alleles and (B) nucleotide diversity (*π*). Figure S3: cross-validation (CV) error plot for ADMIXTURE analyses with *K*-values ranging from 1 to 26, based on 126 individuals and 22 019 SNPs. The CV error decreases sharply with increasing *K* up to *K* = 3, after which it increases. Figure S4: geographical distribution of genetic clusters (*K* = 3) from ADMIXTURE analysis, based on 126 individuals and 22 019 SNPs of *Abies alba.*  Figure S5: Stairway plot of *Abies alba* demographic history showing changes in effective population size (*N*_e_) over the last 30 000 years.
