# Supplementary figures and images for: Postglacial genetic legacies and climate-driven demography inform conservation of silver fir
Source: Ann Bot. 2026 Feb 9;137(7):2082–94. doi: 10.1093/aob/mcag029 (PMC13319518; doi:10.1093/aob/mcag029)

**SUPPLEMENTARY DATA**

**Figure S1**


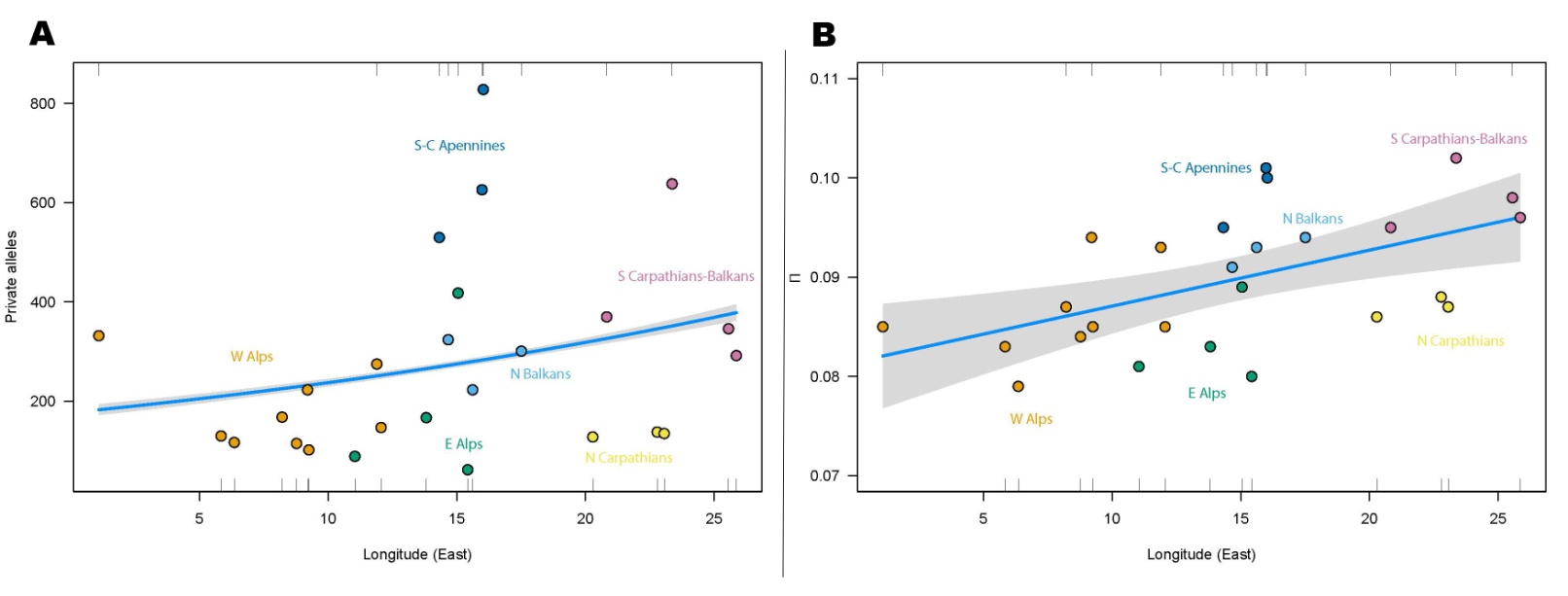


**Figure S2**


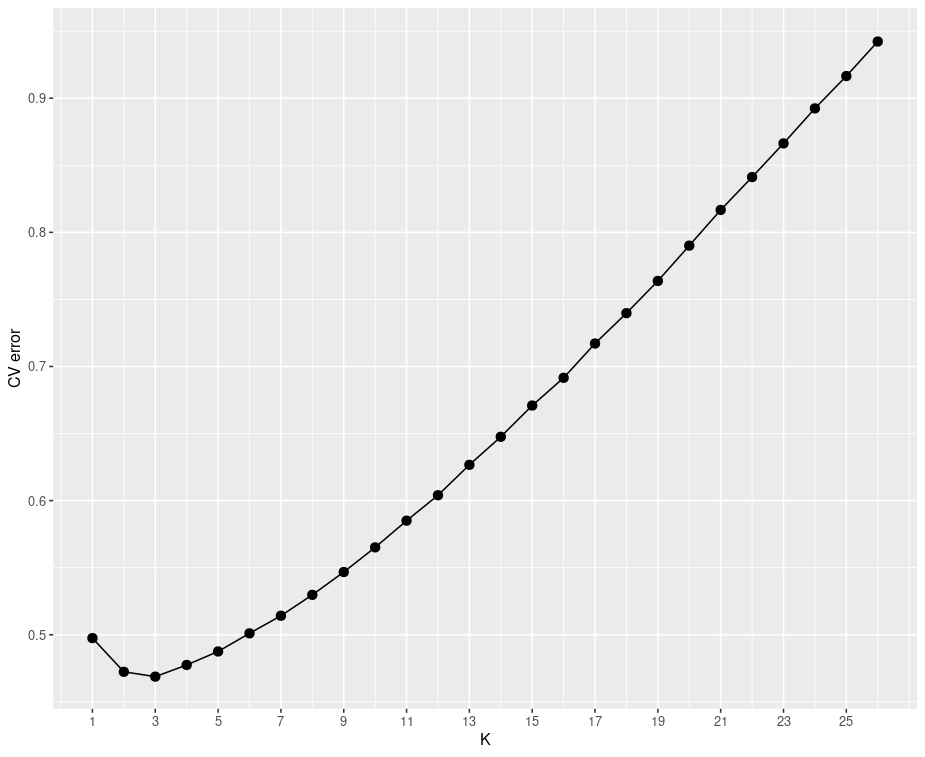


**Figure S3**

**Figure S4**


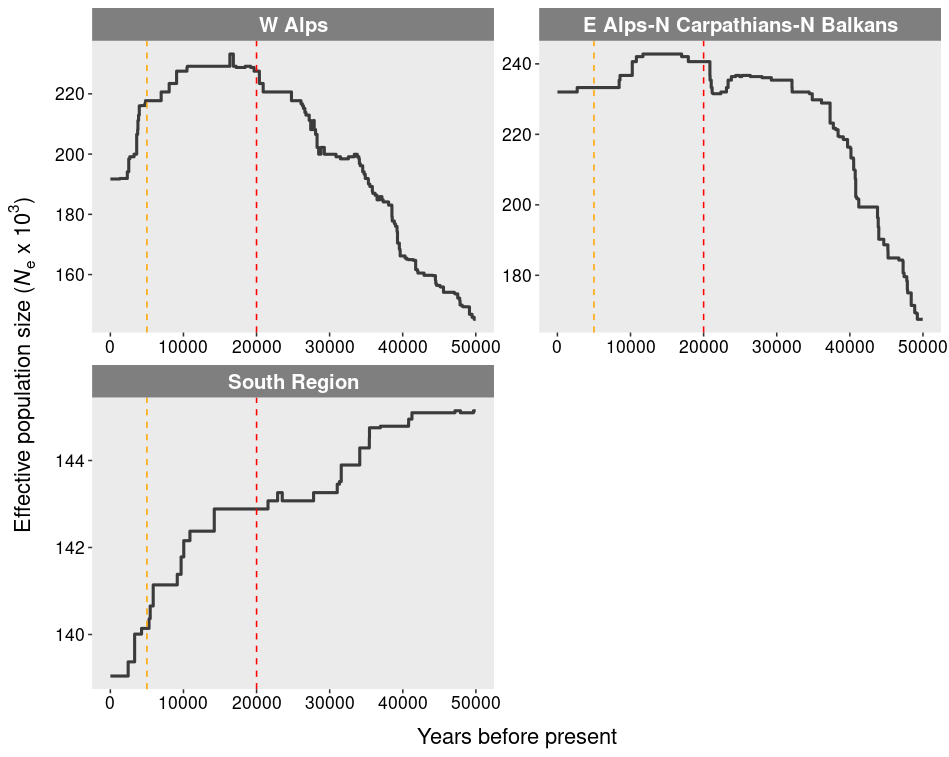


**Figure S5**

Supplement: mcag029_Supplementary_Data [file mcag029_supplementary_data.docx]
